# Evidence of Mitochondrial Dysfunction in Autism: Biochemical Links, Genetic-Based Associations, and Non-Energy-Related Mechanisms

**DOI:** 10.1155/2017/4314025

**Published:** 2017-05-29

**Authors:** Keren K. Griffiths, Richard J. Levy

**Affiliations:** Department of Anesthesiology, Columbia University Medical Center, New York, NY, USA

## Abstract

Autism spectrum disorder (ASD), the fastest growing developmental disability in the United States, represents a group of neurodevelopmental disorders characterized by impaired social interaction and communication as well as restricted and repetitive behavior. The underlying cause of autism is unknown and therapy is currently limited to targeting behavioral abnormalities. Emerging studies suggest a link between mitochondrial dysfunction and ASD. Here, we review the evidence demonstrating this potential connection. We focus specifically on biochemical links, genetic-based associations, non-energy related mechanisms, and novel therapeutic strategies.

## 1. Introduction

Autism spectrum disorder (ASD) describes a group of neurodevelopmental disorders characterized by impaired social interaction and communication as well as restricted and repetitive behavior [1]. The diagnostic criteria have recently been modified in the Diagnostic and Statistical Manual of Mental Disorders (DSM V) and require each of the following for diagnosis: persistent deficits in social and emotional reciprocity, impairments in nonverbal communication, and abnormalities in establishing relationships with peers [1]. Secondarily, to be diagnosed with autism, patients must display at least two of the following: stereotypical and repetitive motor or verbal behavior, excessive or repetitive adherence to routines and patterns of behavior, highly restricted and overly fixated interests, or exaggerated or hyporeactive responses to sensory input [1]. Finally, symptoms must manifest early in childhood and impair day-to-day functioning [1].

ASD is the fastest growing developmental disability in the United States and approximately 1 in 68 children carry the diagnosis [2, 3]. Males are affected 4 to 5 times more commonly than females and the prevalence has increased 10 to 17% each year over the last several years [2, 3]. There is currently no cure for autism and medical therapy is limited to targeting behavioral symptoms [4]. Although the underlying cause of autism is unknown, the most promising hypotheses suggest genetic predisposition, epigenetic modifications, nutritional influences, and exposure to environmental toxins at critical periods during development [5, 6]. A growing body of clinical, genetic, and biochemical evidence now suggests that ASD, or at least a subset of ASDs, may also be linked to impaired mitochondrial function [7].

Mitochondria are organelles primarily responsible for aerobic energy production in vertebrate eukaryotic cells [8]. In addition, they also play an important role in calcium homeostasis and signaling, regulation of apoptosis, and reactive oxygen species (ROS) formation [9]. Because the central nervous system (CNS) accounts for 20% of the body's metabolic demand and developing neurons depend on oxidative phosphorylation for critical developmental processes, the immature brain is uniquely vulnerable to defects in bioenergetic capacity [8, 10, 11]. Thus, it is not surprising that emerging studies suggest that mitochondrial impairments may contribute to or cause a variety of neurodevelopmental disorders [10]. Here, we review the evidence demonstrating a potential connection between mitochondrial dysfunction and autism. We focus specifically on biochemical links, genetic-based associations, non-energy related mechanisms, and novel therapeutic strategies.

## 2. The Biochemical Link between Mitochondrial Dysfunction and Autism

In 1985, Coleman and Blass observed elevated levels of lactate in the plasma of four patients with autism, suggesting a defect in oxidative phosphorylation [12]. However, it was not until 1998 that the concept of autism as a mitochondrial disease was first proposed [13]. This hypothesis was based on finding lactic acidosis, elevated urine levels of Krebs cycle metabolites, plasma carnitine deficiency, and decreased brain glucose utilization and adenosine triphosphate (ATP) levels in autistic patients [13]. Over the last 30 years, numerous reports have corroborated the notion of bioenergetic deficiency in children with ASD by detecting a variety of abnormal biomarkers in the brain, plasma, cerebral spinal fluid (CSF), urine, fibroblasts, skeletal muscle, and buccal mucosa [7, 11, 14]. In this section, we present the evidence of a potential biochemical link between impaired mitochondrial function and ASD.

### 2.1. Indirect and Direct Evidence from Non-CNS Tissue

Defects in oxidative phosphorylation are known to result in lactic acidemia, abnormal lactate: pyruvate ratios, accumulation of alanine, and increased acyl-carnitine levels in the plasma and urine [7]. A number of investigators have identified such indirect evidence of mitochondrial dysfunction in a variety of peripheral tissues and samples obtained from autistic children [14]. For example, in a study of 60 autistic patients aged 2 to 40 years of age, 8.3% of them demonstrated biochemical markers of abnormal aerobic respiration [7]. These included elevated plasma lactate and alanine levels and the presence of organic acids in the urine such as 3-methyl-glutaconic acid, citric acid cycle intermediates, and dicarboxylic acids [7]. In other work, 20% of children with ASD had elevated plasma lactate levels along with increase lactate: pyruvate ratios [15]. Further evidence included reduced total and free serum carnitine levels, decreased pyruvate, and increased alanine and ammonia in a cohort of patients with a diagnosis of ASD [16]. In a retrospective review of the medical records from 25 children with autism, 76% had elevated blood lactate, 53% had increase pyruvate levels, 20% demonstrated an increase lactate: pyruvate ratio in fibroblasts, and 42% displayed abnormal urine organic acid analysis [17].

As far as direct evidence of abnormal electron transport chain (ETC) function in peripheral tissues, Graf et al. reported pathologically increased complex I activity in mitochondria isolated from a skeletal muscle biopsy of a patient with autism [18]. On the other hand, defects in complex I, III, IV, and V were identified in skeletal muscle mitochondria obtained from a cohort of autistic children who also exhibited hypotonia, epilepsy, and developmental delay [19]. In other work, two children diagnosed with autism and a chromosome 15q11-q13 inverted duplication demonstrated decreased skeletal muscle complex III activity [20]. Further evidence of impaired ETC activity was described in another case series of patients with ASD [21]. These patients exhibited impairments in skeletal muscle complexes I, II, II + III, and IV [21]. In the retrospective chart review of 25 autistic children described above, quadriceps muscle, skin fibroblasts, and liver biopsy samples revealed a complex I defect in 64% of patients, a complex II impairment in 8%, a complex III defect in 20% of patients, and depressed complex IV function in 4% of children [17]. In other work, Shoffner and colleagues found deficits in skeletal muscle mitochondria complexes I, I + III, I + III + IV, and V in 28 children co-diagnosed with ASD and mitochondrial disease [22]. In addition, impaired activity of complex I, complex III, and/or complex IV was also described in leukocytes or buccal mucosa obtained from autistic patients in a study published in 2010 [23, 24]. Since then, a number of other publications have corroborated these findings in children with ASD [25–28]. Although, a few studies have demonstrated increased activity of certain ETC complexes, the majority have identified depressed function in autism [14]. As a whole, complex I appears to most frequently affected, followed in descending order by complex IV, complex III, complex V, and complex II [14].

### 2.2. Direct Evidence from Brain Tissue

In work that evaluated postmortem brain samples, investigators found decreased steady-state levels of complexes III and V in the cerebellum, complex I in the frontal cortex, and complexes II, III and V in the temporal cortex in the autistic developing brain versus that of age-matched controls [29]. In addition, markers of oxidative stress were significantly increased in the cerebellum and temporal cortex of children with ASD [29]. Of note, no differences in ETC complex protein expression were detected between groups in the parietal and occipital cortices and no changes were observed in adults with autism. The results suggested mitochondrial impairment in the brains of autistic children who were between the ages of 4 and 10 years, defining a potential window of vulnerability [29].

In follow up work, the investigators demonstrated more than 30% reduction in the activities of complexes I and V, and pyruvate dehydrogenase in the frontal cortex of the postmortem autistic brain [30]. Such defects in complexes I or V activity were identified in 43% of autistic specimens while complex III impairment was found in 29% of autistic brains [30]. Furthermore, 29% of autistic brain samples displayed a combination of abnormal activities involving multiple complexes while 14% demonstrated deficits in all ETC complexes [30]. The authors also identified increased mitochondrial gene copy number [30]. Taken together, the findings provided direct evidence for mitochondrial dysfunction in the developing autistic brain.

In corroborative work, Brodmann area 21 within the lateral temporal lobe of the ASD brain was assessed in postmortem samples [31]. This region of the brain is responsible for auditory processing, language, and social perception and has been implicated in the manifestation of the autistic phenotype [31]. Similar to the prior studies, the researchers identified decreased protein levels of complexes I, III, IV, and V in the autistic brain and impaired complex I and IV activities [31]. They also found reduced levels of superoxide dismutase (SOD) and enhanced oxidative DNA damage [31]. Also, consistent with prior work, much of the mitochondrial abnormalities were identified in younger children (under 10 years of age) indicating vulnerability in the developing autistic brain [31].

These findings were confirmed in yet another postmortem analysis [32]. In this work, investigators reported reduced protein expression of various subunits of complex I, III, IV and V in the motor cortex, thalamus, and cingulate gyrus of the autistic brain compared to controls [32]. Specifically, ATP5A1 (complex V), ATP5G3 (complex V), and NDUFA5 (complex I) were consistently decreased in all brain regions examined. In other work, protein expression of 84 genes important for mitochondrial homeostasis was also evaluated in the postmortem ASD brain [33]. Researchers found decreased expression of many different genes such as *MTX2* (import receptor for mitochondrial pre-proteins), *NEFL* (regulates mitochondrial morphology, fusion, and motility) and *SLC25A27* (mitochondrial uncoupling protein 4) [33]. These gene products are responsible for a wide range of mitochondrial functions and the findings suggest a possible mechanistic role for impaired mitochondria in autism beyond oxidative phosphorylation.

Neuroradiographic imaging has been helpful in identifying metabolic abnormalities in the brain in patients with autism [34]. For example, proton magnetic resonance spectroscopy (^1^H–MRS), a non-invasive imaging modality, permits in vivo quantification of specific markers of brain metabolism such as creatine, phosphocreatine, choline, myo-inositol, lactate and N-acetyl aspartate (NAA) [34]. In addition, phosphorous-31 magnetic resonance spectroscopy (^31^P–MRS), a different, but related technique, enables non-invasive measurement of high-energy phosphates such as adenosine triphosphate (ATP), adenosine diphosphate (ADP), phosphocreatine, and inorganic phosphate within the brain [35]. Such neuroradiographic assessments have identified consistent decreases in brain levels of NAA, creatine, phosphocreatine, choline and myo-inositol in children affected by ASD compared to healthy controls [34, 36–42]. Variable levels of these metabolic markers have been described in different brain regions in adults with ASD [34, 43]. Results from non-invasive measurement of lactate have been less consistent and likely relate to challenges in detecting lactate by these techniques [34, 41]. It should also be noted that interpretation of MRS data has limitations and may be complicated by inconsistencies in methodology and variability in the phenotype of subjects examined [34]. As a whole, however, the data from non-invasive imaging suggests metabolic dysfunction in the autistic brain.

## 3. The Genetic Link between Mitochondrial Dysfunction and Autism

Each mitochondrion has multiple copies of the mitochondrial genome (mtDNA) within its matrix [44]. mtDNA encodes for 13 essential subunits of the ETC enzymes (complexes I, III, IV, and V), 22 transfer RNAs (tRNAs), and 2 types of ribosomal RNA (rRNA) [44, 45]. The remaining ETC complex subunits are encoded by nuclear deoxyribonucleic acid (nDNA) [44, 45]. In addition, mitochondria contain a variety of non-ETC enzymes, membrane proteins, and other molecular components that are necessary for maintaining homeostasis and mitochondrial function [44, 45]. These proteins and enzymes are also encoded by nDNA [44, 45]. Therefore, genetic mutations in either mtDNA or nDNA have the potential to cause mitochondrial dysfunction [44]. It is estimated that 1 in 2000 children born in the United States will develop a genetic-based mitochondrial disease [46]. Of these, 15% result from a mutation in mtDNA while 85% manifest from mutations in nDNA [9].

**Figure 1:**
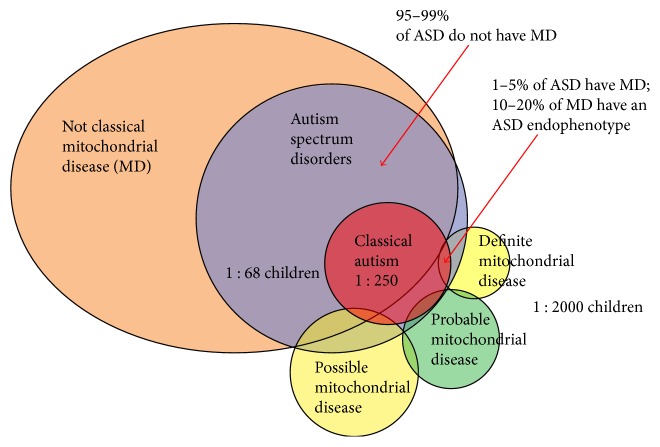
Relationship between mitochondrial disease, ASD, and autism. Mitochondrial disease in most children with ASD is of the non-classical variety. Up to 5% of children with autism have classical mitochondrial disease while 10–20% of patients with classic mitochondrial disease demonstrate ASD features. The co-existence of ASD with mitochondrial disease is higher than the prevalence of either ASD or mitochondrial disease in the general population, suggesting a link between mitochondrial dysfunction and autism. Reprinted from [9].

Defects in mitochondrial function are classified as primary or secondary in nature [7, 9]. Primary mitochondrial defects arise as a direct consequence of gene mutations that impair aerobic ATP synthesis, while secondary mitochondrial dysfunction is characterized by deficits in oxidative phosphorylation that result indirectly from other genetic or metabolic derangements [7, 9]. Because of the high prevalence of ASD, it would be expected that a proportion of patients with an inherited mitochondrial cytopathy also carry a diagnosis of autism [7]. If there was no link between mitochondrial disease and autism, it would be expected that ~1 in 2000 children with ASD would also carry a diagnosis of mitochondrial cytopathy [7]. However, the co-existence of mitochondrial disease in cohorts with ASD is higher than the general population, suggesting a role for mitochondrial dysfunction in autism (Figure 1) [7]. In this section, we review the mitochondrial disease-related genetic abnormalities that have been identified in autistic children.

### 3.1. Mitochondrial DNA Abnormalities

Defects in mtDNA have been demonstrated in children in which ASD and mitochondrial disease co-exist [7, 14]. Although many of these mtDNA mutations and mitochondrial diseases are well known and have been thoroughly classified, some are poorly understood and have yet to be characterized. For example, mutations of mtDNA were identified in a unique cohort of children that suffered from hypotonia, epilepsy, autism, and developmental delay (HEADD) [19]. Although this group of patients could not be placed into a previously described category of mitochondrial disease, they displayed autistic features and ~50% harbored large-scale mtDNA deletions [19]. A limitation of such observations, in general, is that conclusions regarding cause and effect cannot be made. Thus, with many of these reports, only an association between mtDNA mutation and autism can be inferred. However, some studies suggest a functional contribution of mitochondrial disease to the autistic phenotype [22]. For example, in a retrospective analysis of 28 patients co-diagnosed with ASD and mitochondrial disease, autistic regression occurred with fever in 71% of those who regressed versus regression without fever in 29% [22].

One of the best described mitochondrial diseases, mitochondrial encephalomyopathy, lactic acidosis, and stroke-like episodes (MELAS), is also known to co-exist with autism. MELAS results commonly from the A3243G mtDNA mutation and this genetic defect has been shown to be associated with autism [21, 47]. In 1999, Sue et al. published a report of three children with the MELAS A3243G mutation and infantile encephalopathy [47]. One of these children went on to develop autistic features later in life [14]. Subsequently, Pons et al. analyzed 5 autistic children who had a family history of mitochondrial disease [21]. Two of these patients harbored the A3243G mutation while, in two others, the A3243G mutation was identified in maternal samples [21]. The fifth child in this study was found to have mtDNA depletion syndrome [21].

Another early report connecting a mtDNA mutation with ASD was published in 2000 [18]. In this work, Graf et al. reported on two siblings who carried a point mutation (G8362A) in the mitochondrial tRNA for lysine [18]. The 6 year-old sister was diagnosed with Leigh syndrome after developing ataxia and myoclonus at 15 months of age [18]. She was dysarthric, had a moderate intellectual disability, and demonstrated a complex IV defect in her skeletal muscle [18]. Her younger brother was diagnosed with ASD following a regression of developmental milestones between 1.5 to 2 years of age [18]. He carried the same mtDNA mutation as his sister, yet at a lower level of heteroplasmy [18]. By 3.5 years of age, he was hyperactive, lacked verbal communication, and exhibited self-injurious behavior [18]. Unlike his sister, his skeletal muscle biopsy demonstrated hyperactivity of complex I [18].

In another study, the records of 25 patients with a primary diagnosis of ASD were reviewed [17]. Twenty-one of these children met criteria for definitive mitochondrial disease and four met criteria for probable mitochondrial disease [17]. Whole mitochondrial genome sequencing was performed in 11 patients and 16 underwent selected mitochondrial mutation analysis [17]. They identified mtDNA mutations in 7 of these cases [17]. Three mutations occurred in highly conserved regions of the mitochondrial genome, known to encode for the mitochondrial tRNA^leu^ and the ND1 and ND4 subunits of complex I [17]. These mutations were interpreted as likely having functional sequelae given prior characterization of the tRNA^leu^ mutation, that the patient with the ND1 variant demonstrated reduced muscle complex I activity and an increased fibroblast lactate: pyruvate ratio, and that a missense mutation of ND4 had previously been reported in a patient with Leigh syndrome [17, 48, 49]. The four remaining mutations were of unclear functional significance [17].

In 2010, Giulivi and colleagues analyzed mtDNA from the leukocytes of 10 children, aged 2 to 5 years, who were diagnosed with ASD [23]. This was a subanalysis of the Childhood Autism Risk from Genes and Environment (CHARGE) study [23]. Five children with autism had an increased mtDNA copy number compared to controls and 2 of the 10 autistic children had mutations in the cytochrome b gene segment which was associated with over replication [23]. In another subset of patients from the CHARGE study, Napoli and colleagues compared 67 autistic children with 46 controls [50]. The investigators assessed for mtDNA mutations of *CYTB,* the gene that encodes cytochrome b and *ND4,* the gene that encodes the ND4 subunit of complex I [50]. They found that children with autism had a significantly higher incidence of mutation of these two genes compared to controls [50]. Fifteen percent of patients with ASD harbored an *ND4* deletion while 21% had a mutation of *CYTB* compared to 7% and 9% of healthy children, respectively [50].

Recently, whole exome sequencing from 903 ASD proband-mother-sibling trios demonstrated that autistic children were 53% more likely to have heteroplasmic mutations in non-polymorphic sites (regions more likely to produce deleterious effects in oxidative phosphorylation) than unaffected siblings [51]. Autistic individuals also had 1.5 times as many non-synonymous mutations and 2.2 times as many predicted pathogenic mutations than non-autistic siblings [51]. These findings were in contrast to an earlier report in which whole mitochondrial genome sequencing of ~400 autistic children failed to identify evidence of an association between mitochondrial mutations and autism using the proband's father as a control [52].

### 3.2. Nuclear DNA Gene Defects

As discussed above, defects in nuclear expression of mitochondrial proteins can impair oxidative phosphorylation and mitochondrial homeostasis. In fact, the majority of inherited mitochondrial diseases occur due to mutations in nDNA [9]. Thus, if mitochondrial disease and dysfunction cause autism, there should be evidence of an association between nDNA mutations and ASD. In 2003, Filipek et al. reported two cases of children diagnosed with autism who also had chromosome 15q11-q13 inverted duplication [20]. Both children exhibited mitochondrial dysfunction with decreased complex III activity and marked mitochondrial hyperproliferation in skeletal muscle [20]. Although the mechanism of a 15q11-q13 defect is unknown, it is possible that the gene product is involved in complex III regulation [20].

In work that investigated a link between potential candidate genes in the 2q24-q33 region and autism, Ramoz and colleagues identified two single nucleotide polymorphisms (SNPs) (rs2056202 and rs2292813) in the *SLC25A12* gene [53]. The *SLC25A12* gene is associated with neurite development, encodes the calcium-dependent mitochondrial aspartate/glutamate carrier (AGC1) that is known be functionally important in metabolically active neurons, and has been shown to be upregulated in the autistic prefrontal cortex [14, 54]. Ramoz and colleagues found that 48% of autistic patients harbored these SNPs in the *SLC25A12* gene [53]. Although these findings have been corroborated by other investigators, it should be noted that some studies have demonstrated discrepant results [55–58].

Candidate genes for mitochondrial proteins that may contribute to the autistic phenotype have also been explored in other regions of nDNA. For example, Marui et al. evaluated the 7q32 region and studied a cohort of 235 Japanese patients [59]. The investigators identified two SNPs (rs12666974 and rs23779262) in the *NDUFA5* gene which were associated with ASD. *NDUFA5* encodes NADH–ubiquinone oxidoreductase 1 alpha subcomplex 5, an accessory subunit of complex I of the ETC [60]. Thus, such mutations could have consequences for complex I activity. In other research, a 1 Mb deletion was identified in the 5q14.3 region in a 12 year-old child with autism, mitochondrial disease, cognitive impairment, and dysmorphic features [24]. Buccal mucosa obtained from the patient demonstrated severely decreased complex IV activity and mildly reduced complex I activity [24]. The authors suggested that the gene product likely regulates expression or assembly of subunits of complexes I and IV [24].

In a recent meta-analysis, Rossingol and Frye attempted to determine the prevalence of genetic abnormalities in autistic children with mitochondrial disease [11]. They identified 18 publications that described a total of 112 children diagnosed with both ASD and mitochondrial disease [11]. Symptoms and signs attributable to classic mitochondrial disease in these children were similar to the general population with mitochondrial disease, however, were significantly higher than the general autistic population [11]. This indicated that the cohort in which ASD and mitochondrial disease co-exist represented a distinct subgroup of children [11]. Importantly, they found that only 21% of patients in this cohort had mutations in mtDNA or nDNA or chromosomal abnormalities [11]. Thus, the majority was not directly associated with known genetic abnormalities, suggesting a role for secondary mitochondrial dysfunction [11]. So, although genetic mutations have been described in children with autism and mitochondrial disease, the role of such abnormalities in the ASD phenotype, whether causative or associative, is not fully known.

## 4. Limitations of the Presented Studies

Although the literature suggests potential biochemical and genetic links between impaired mitochondrial function and ASD, there are limitations that should be noted. First, it is not possible to determine if mitochondrial dysfunction causes ASD or results from autism or other associated processes. A major confounding factor is the presence of comorbidities in this patient population [61]. Epilepsy, cerebral palsy, and non-mitochondrial genetic syndromes, for example, can secondarily affect mitochondrial function [61]. Thus, interpretation of mitochondrial-based research in autistic subjects can be challenging in the context of such comorbid states. Next, studies of patients with ASD are prone to selection bias and many of the investigations fail to report the lack of an association between mitochondrial impairment and autism [61]. In addition, small sample sizes limit interpretation and the generalizability of many of these studies [61].

When considering measures of metabolism, biological tissue and sample preservation and handling are critical factors necessary for proper data interpretation [61]. For example, false positive values can result from imperfect technique and methodology [61]. Unfortunately, many of the published studies did not adequately detail exactly how samples were handled or processed [61]. Another limitation is the use of lymphocytes, fibroblasts, skeletal muscle, or buccal mucosa as surrogates for brain tissue in order to detect mitochondrial defects [61]. Defects in such peripheral samples do not necessarily indicate CNS disease. In addition, analyzing lymphocytes may be specifically problematic given that children with ASD can have altered immunological function and mitochondria within inflammatory cells may be indirectly affected [61]. However, despite these limitations, there is general agreement in the research community that the evidence suggests an association between mitochondrial disease and autism [61].

## 5. Alternative Mechanisms of ASD Pathogenesis

In addition to producing aerobic energy, mitochondria also regulate calcium signaling, mediate apoptosis, and generate ROS [9]. Such processes are important for normal brain development. Thus, mitochondrial dysfunction has the potential to affect the immature brain via non-energetic pathways. It has been hypothesized that ASD may manifest from oxidative stress, immune dysfunction, or defects in calcium homeostasis [7, 14, 62]. Therefore, in this section we will summarize the evidence that supports these alternative mitochondria-related mechanisms of ASD.

### 5.1. Oxidative Stress and Abnormal Redox Regulation

The mitochondrion is a major source of ROS produced within the cell [63–65]. Superoxide, the principal free radical formed within mitochondria, is generated by complexes I and III as a by-product of oxidative phosphorylation [66, 67]. Low levels of ROS are known to be required for physiological signaling and homeostasis and play a critical role in processes such as the regulation of vascular tone, erythropoietin production, and programmed cell death [66]. However, when generated pathologically, ROS can irreversibly damage DNA, cellular proteins, and membrane lipids [66]. Such oxidative stress has been implicated in a variety of neurodegenerative disease states [68].

To counterbalance ROS toxicity and to provide cytoprotection, cells are equipped with a variety of antioxidants such as glutathione (GSH), SOD, glutathione peroxidase, catalase, ascorbic acid, *α*-tocopherol, and *β*-carotene [66]. In addition, mitochondria contain their own antioxidant enzymes such as manganese-dependent superoxide dismutase (MnSOD) in the mitochondrial matrix and copper-zinc superoxide dismutase (CuZnSOD) in the intermembrane space [69, 70]. Thus, endogenous antioxidants are important to enable the cell to strike a balance between superoxide formation and aerobic energy production in order to prevent oxidative stress and cellular damage. Excess ROS formation and oxidant injury can result from impaired ETC activity, defects in antioxidant content and function, or their combination [71]. Furthermore, free radicals can target and alter respiratory chain integrity, leading to further superoxide production [71, 72]. Thus, mitochondrial dysfunction can cause oxidative stress and result from it as well.

A number of studies have found that individuals with ASD display hallmarks of increased oxidative stress or abnormalities in redox regulation, supporting the notion of a mechanistic role for ROS in the manifestation of the autistic phenotype [7, 14, 62]. Such evidence of increased oxidative damage to DNA, proteins and lipids has been identified in blood, urine, and post-mortem brain samples from autistic individuals [62]. For example, markers of impaired capacity for methylation and enhanced oxidative stress, such as lower S-adenosylmethionine-to-S-adenosylhomocysteine ratios and lower redox ratios of reduced glutathione-to-oxidized glutathione (GSH/GSSG), have been found in the plasma of children with ASD [73, 74]. With regard to biomarkers in urine, elevated levels of the lipid peroxidation biomarker, 8-isoprostane-F_2*α*_, have been detected in autistic children [75]. Further investigation corroborating these results found increased urinary levels of isoprostane F_2*α*_-VI, 2,3-dinor-thromboxane B_2_ (a marker of platelet activation), and 6-keto-prostaglandin F_1*α*_ (a marker of endothelial activation) in 26 children with ASD [76]. In another study, plasma levels of malondialdehyde (a marker of fatty acid peroxidation) were found to be significantly increased in children with ASD and were associated with a concomitant decline in levels of *α*-tocopherol and GSH [77]. Decreased levels of other antioxidant enzymes, such as erythrocyte SOD, erythrocyte and plasma glutathione peroxidase, serum transferrin, and serum ceruloplasmin have also been described in autism [78, 79]. Importantly, a correlation between such reduced levels and loss of language skills has been established in children with ASD [79].

Post-mortem analyses have more directly demonstrated abnormalities in enzymes involved in redox homeostasis and have identified evidence of oxidative damage to proteins, lipids, and DNA within the autistic brain [31, 62, 78, 80]. For instance, decreased MnSOD activity and increased 8-hydroxy-2′-deoxyguanosine, a marker of oxidatively modified DNA, were identified in Brodmann area 21 within the temporal lobe of autistic subjects [31]. Gu et al. found decreased activity of glutathione peroxidase, glutathione-S-transferase, and glutamate cysteine ligase in the ASD cerebellum [80]. In addition, investigators have demonstrated decreased levels of reduced GSH, increased levels of GSSG, and lower GSH/GSSG ratios in the cerebellum, temporal lobe, and Brodmann area 22 of individuals with ASD [81, 82]. Evidence of lipid peroxidation has been reported in different language areas of the brain, the cerebellum, hippocampus, and temporal cortex of autistic patients while increased levels of 3-nitrotyrosine, a marker of protein oxidation, have been identified in the cerebellum, orbitofrontal cortex, Wernicke's area, cerebellar vermis, pons, and Brodmann area 22 [29, 83–87].

Thus, autism appears to be associated with a pro-oxidant state. Although mitochondrial dysfunction could certainly cause oxidative stress, the etiology of ASD-related oxidant injury and mechanisms of reduced anti-oxidant defense systems remains unclear. Furthermore, it is unknown if brain-specific oxidative stress is important for the manifestation of symptoms in autism or if generalized redox imbalance is a contributor to the disease phenotype. These are obvious questions for future investigation.

### 5.2. Immune Dysfunction and Inflammation

The inflammatory response and resolution of inflammation are necessary processes for maintaining cellular and tissue homeostasis [88]. In contrast, however, an impaired immune system along with pathological or persistent inflammation can result in disease. In the brain, unchecked neuroinflammation and failure of its resolution can lead to neuropathology and neurodegeneration [88]. Evidence of both inflammation and immune system dysregulation has been identified in the autistic brain and in CSF obtained from subjects with ASD, suggesting a mechanistic role [62].

In 1977, Stubbs and Crawford evaluated the host cellular immune system in 12 children diagnosed with autism [89]. They reported decreased response to phytohemagglutinin in lymphocytes obtain from affected children, suggesting impaired defense mechanisms in autism [89]. More recent work has demonstrated decreases in circulating CD4+ T cells, natural killer cell activity, and Th1/Th2 helper cell ratios in subjects with ASD as well as abnormal accumulation of T lymphocytes in tissues such as the gastrointestinal tract [90–93]. On the other hand, elevated levels of pro-inflammatory cytokines have been identified in postmortem brain tissue, CSF, plasma, and even amniotic fluid of ASD patients [94–101]. Importantly, multiple postmortem studies and one in vivo evaluation that employed positron emission tomography (PET) imaging demonstrated marked neuroinflammation in multiple brain regions in individuals with ASD as evidenced by increased activation of microglia and astroglia [99, 102, 103].

Mitochondria are known to play a role in innate and adaptive immune responses, inflammation, and signaling in response to infection [14, 104]. However, it is unknown how mitochondrial dysfunction affects the immune system or the inflammatory response in autism. Furthermore, it is unknown if such mitochondrial impairments cause autism-associated neuroinflammation and if such inflammation within the developing brain contributes to the ASD phenotype. These are questions that will need to be answered with future investigation.

### 5.3. Abnormal Calcium Homeostasis

Calcium is a ubiquitous second messenger, involved in a variety of cell signaling pathways [105–107]. Because of its central importance to cellular viability, calcium has the potential to adversely affect a wide range of cellular processes when its homeostasis is disrupted. Since calcium is not metabolized, activation and termination of intracellular calcium signaling relies on tight regulation of local calcium concentrations [105–107]. Mitochondria are known to play a vital role in calcium handling within the cell [108]. For example, in response to elevations in cytosolic calcium, mitochondria serve as high-capacity sinks and increase calcium uptake in order to buffer cytosolic levels [108, 109]. This process is carried out by a number of mitochondrial calcium transporters [109].

Oxidative phosphorylation is sensitive to calcium and accumulation within mitochondria is known to stimulate aerobic ATP production by the ETC [108, 109]. Mitochondrial calcium overload, however, can collapse the electrochemical proton gradient, leading to bioenergetic failure and necrotic cell death [108]. Calcium is also known to play a role in mitochondria-mediated apoptotic cell death by inducing opening of the permeability transition pore and increasing ROS following binding to cardiolipin on the inner mitochondrial membrane [105, 110]. Thus, defects in calcium homeostasis can result in mitochondrial dysfunction, oxidative stress, and cytotoxicity.

Neurotransmitter-mediated calcium signaling is important for the recruitment and accumulation of mitochondria to postsynaptic regions, a process that is critical for neuronal calcium buffering and synapse strength [111]. Calcium signaling is important for neurotransmitter release from presynaptic neurons as well as signaling in postsynaptic neurons in response to neurotransmitters such glutamate and *γ*-aminobutyric acid (GABA) [111, 112]. Glutamate receptors are ligand-gated calcium channels while GABA receptors trigger calcium influx via voltage-gated calcium channels [112]. Calcium transients evoked by GABA, principally an excitatory neurotransmitter during neurodevelopment, are necessary for the critical processes of brain development [112]. Thus, aberrant calcium homeostasis could interfere with proliferation, migration, dendritic arborization, Purkinje cell development, synapse formation and maintenance, and cell death [111, 112]. Furthermore, defects in calcium signaling could be further compounded by mitochondrial dysfunction and can result in decreased neurotransmitter signaling, especially in neurons that have high firing rates of firing [112, 113]. This could explain the relative increase in excitatory-to-inhibitory neuron ratio observed in patients with ASD [114].

Finally, calcium is impacted by ATP-mediated neuronal purinergic signaling [111]. Perisynaptic ATP binds to astrocyte receptors, leading to calcium release from mitochondria, depolarization of the mitochondrial membrane potential, and generation of ROS [111]. In addition, extracellular ATP binds to microglial purinergic receptors, resulting in an increase in intracellular calcium, activation of microglia, neuroinflammation, and cell death [9, 111]. Thus, aberrancies in calcium signaling could account for oxidative stress and neuroinflammation observed in ASD.

Abnormalities in the expression of a number of genes involved in calcium signaling or homeostasis have been associated with autism [14, 112, 115]. These include *ATP13A4*, *ATP2B2*, *CACNA1C*, *CACNA1F*, *CNCNA1H*, *KCNMA1*, *IL1RAPL1*, *NCS1*, *CAPS2,* and *SLC25A12* [14, 112, 115]. The calcium-dependent mitochondrial aspartate/glutamate carrier, AGC1, and the gene that encodes it (*SLC25A12*) are of particular interest because they link defects in calcium regulation with mitochondrial dysfunction [115]. For example, expression of *SLC25A12* was found to be decreased in the motor cortex and cingulate gyrus and increased in the prefrontal cortex of autistic individuals [54]. Other work identified elevated neocortical calcium levels as the cause of increased AGC1 activity in the ASD brain [116]. Importantly, the authors also identified as association between abnormal calcium signaling in autism with pathologically increased cytochrome oxidase activity and oxidative stress [116]. Thus, increased AGC1 transport activity may result in mitochondrial dysfunction and ROS formation. Because defects in calcium homeostasis can cause or result from disturbances in mitochondrial function, it is possible that impaired calcium signaling plays a role in the connection between mitochondrial dysfunction and autism.

## 6. Treatment of ASD

There is currently no cure for autism because the underlying cause of ASD is unknown. Therefore, medical management has been limited to therapies that address the behavioral symptoms [1]. Intensive behavioral intervention, implemented early in life, remains the widely accepted standard of care for children with autism [117, 118]. However, the accumulating evidence of metabolic abnormalities associated with ASD provides insight into potential mechanisms of disease and has elucidated novel candidate targets for therapeutic intervention [117, 119]. Here we review the different experimental approaches that target mitochondrial dysfunction and oxidative stress in autism and have been trialed in an effort to restore mitochondrial homeostasis and improve the clinical manifestations of ASD.

### 6.1. Targeting Mitochondrial Dysfunction and Oxidative Stress

Dietary supplements, like those typically used for the treatment of mitochondrial diseases, have been used to treat children with ASD [117, 119]. Such supplements include L-carnitine, coenzyme Q10, ubiquinol, B vitamin-containing multivitamins, ascorbic acid, *α*-tocopherol, and N-acetyl-L-cysteine [117, 119]. Treatment with L-carnitine, an essential nutrient important for the fatty acid transport across the mitochondrial membrane, was shown to improve core and associated ASD symptoms in a number of controlled trials [119–121]. In one of these investigations, serum carnitine levels were found to correlate with cognitive and behavioral scores [121].

In other work, supplementation with antioxidants such as N-acetyl-L-cysteine (a precursor to glutathione), coenzyme Q10, ubiquinol, ascorbic acid, *α*-tocopherol, methylcobalamin, and carnosine also improved behavioral symptoms associated with autism [122–129]. In a randomized double-blind placebo controlled trial, a formulation of multivitamins combined with mineral supplements (containing multiple mitochondrial cofactors, vitamins, and antioxidants) improved plasma or erythrocyte levels of methylation, glutathione, oxidative stress, sulfation, ATP, nicotinamide adenine dinucleotide (NADH), and nicotinamide adenine dinucleotide phosphate (NADPH) and improved overall behavior, hyperactivity, tantrums, and receptive language in children and adults with ASD [126, 127]. Trials involving other antioxidants such as the phytochemical sulforaphane and the flavonoid luteolin also improved ASD symptoms, however no metrics of oxidative stress were examined [130, 131].

### 6.2. Other Metabolic Targets

Folic acid is important for redox metabolism, methylation, and mitochondrial homeostasis [132, 133]. Disruption of folate receptor *α* activity occurs in autism due to autoantibodies and mitochondrial dysfunction and results in CNS folate deficiency [134]. Severe reductions in cerebral folate levels can lead to neurodevelopmental regression and the autism phenotype [119]. Importantly, targeted treatment with folinic acid has been shown to partially or completely improve communication, social interaction, attention, and stereotypical ASD behavior in patients with autoantibodies to folate receptor *α* [135–137]. Thus, targeting various causes and effects of mitochondrial dysfunction in autism may rescue behavior and minimize the clinical manifestations of ASD.

## 7. Conclusion

The literature reviewed here suggests a link between abnormalities in mitochondrial homeostasis and ASD and provides biochemical and genetic evidence to support a role for mitochondrial dysfunction in the pathogenesis of the autism phenotype. Mechanistically, the connection may involve defects in bioenergetic capacity as well as non-energy related pathways. However, it is not clear if mitochondrial impairments cause ASD or if they are merely associated with the disease process. Positive patient behavioral responses to conventional mitochondrial disease therapies are promising, however, further investigation is necessary. Future work should focus on determining how mitochondrial dysfunction causes the autistic phenotype as well as how defects in mitochondrial homeostasis predispose individuals to ASD via interaction with environmental toxins, dietary factors, and epigenetic modifications during critical periods of development. Establishing a causative relationship between mitochondrial dysfunction and ASD and elucidating the exact mechanisms will permit the development of more precisely targeted therapies in the future. Ultimately, with improved knowledge and innovation, we may one day be able to prevent or cure autism.
